# Synthesis and Performance of Epoxy-Terminated Hyperbranched Polymers Based on Epoxidized Soybean Oil

**DOI:** 10.3390/molecules30030583

**Published:** 2025-01-27

**Authors:** Guang-Zhao Li, Qiuhong Wang, Chongyu Zhu, Shuai Zhang, Fumei Wang, Lei Tao, Youqi Jiang, Qiang Zhang, Wenyan Wang, Rui Han

**Affiliations:** 1 Key Laboratory of Materials and Surface Technology (Ministry of Education), School of Materials Science and Engineering, Xihua University, Chengdu 610039, China; coriander180716@163.com (Q.W.); brucezhang.scu@foxmail.com (S.Z.); fumei_wang0919@163.com (F.W.); j13990553070@163.com (Y.J.); wwyandmmy@163.com (W.W.); ruihan_harry@163.com (R.H.); 2 College of Chemistry and Chemical Engineering, Donghua University, Shanghai 201620, China; czhu@dhu.edu.cn; 3 The Key Laboratory of Bioorganic Phosphorus Chemistry & Chemical Biology (Ministry of Education), Department of Chemistry, Tsinghua University, Beijing 100084, China; 4 Institute of Polymer Ecomaterials, School of Environmental and Biological Engineering, Nanjing University of Science and Technology, Nanjing 210094, China; zhangqiang@njust.edu.cn

**Keywords:** epoxidized soybean oil, hyperbranched polymers, ring-opening polymerization, degree of branching, adhesion properties

## Abstract

Epoxy-terminated hyperbranched polymers (EHBPs) are a class of macromolecular polymers with a hyperbranched structure containing epoxy groups. They possess characteristics such as low viscosity, high functionality, and thermal stability, which endow them with broad application potential in materials science and chemical engineering. This study uses epoxidized soybean oil (ESO) as the raw material, which undergoes ring-opening reactions with glycerol and is esterified with 2,2-bis(hydroxymethyl)propionic acid (DMPA) to obtain epoxy soybean oil polyol (EGD) with a high hydroxyl value. Subsequently, four types of EHBPs are synthesized by incorporating epichlorohydrin (ECH) in mass ratios of 1:3, 1:4, 1:5, and 1:6 under strong alkaline conditions. The product structure is characterized using FT–IR and GPC. The degree of branching of EGD is calculated using ^1^H NMR and ^13^C NMR spectroscopy. The epoxy value of EHBPs is tested using the hydrochloric acid–acetone method, and the water contact angle, adhesion properties, rheological properties, and thermal properties of the EHBPs are also evaluated. The results show that the degree of branching of EGD is 0.45. The epoxy values of the EHBPs are 0.73, 0.79, 0.82, and 0.89 mol/100g, respectively. As the epoxy value and molecular weight of the epoxy hyperbranched polymers (EHBPs) increase, the water contact angle and adhesion strength of the EHBPs rise progressively and the viscosity decreases. Additionally, the glass transition temperature increases with the increase in the epoxy value. These epoxy hyperbranched polymers with low viscosity and high adhesion strength offer a promising approach for modifying surface coatings or formulating adhesives.

## 1. Introduction

Hyperbranched polymers (HBPs) are a class of highly branched macromolecules with a three-dimensional dendritic structure, characterized by the low degree of chain entanglement, low viscosity, and high functionality [1,2,3,4,5]. Currently, significant progress has been made in the synthesis and application research of HBPs, with a variety of synthetic methods now available, including the self-condensing copolymerization of ABx monomers (x > 1), self-condensing vinyl polymerization, and multi-branched ring-opening polymerization [6,7,8,9]. These methods have enabled the production of HBPs with diverse structures and chemistries. HBPs have been applied in various fields, including polymer membranes, coatings, and biomedical materials [10,11,12].

Studies have indicated that incorporating a substantial number of branching units into HBPs can significantly alter their physical properties. EHBPs are a case in point, where epoxy groups are introduced at the termini of HBPs [13]. The basic structure of these polymers is composed of two main components: the core, which forms the hyperbranched polymer backbone, and the terminal groups featuring epoxy functionalities. The core can be constituted by various types of hyperbranched polymers, such as hyperbranched polyesters [14,15], polyethers, and polyamides [16,17]. Due to the diversity in core structures, hyperbranched epoxy compounds exhibit a range of distinct physical and chemical properties. The introduction of epoxy groups enhances the physical and chemical properties of HBPs; for example, with the increase in the number of epoxy groups, the adhesive property is enhanced, the water contact angle is increased, and the hydrophilicity is decreased.

The synthesis of hyperbranched epoxy polymers typically involves two main stages: the preparation of hyperbranched polymers and the subsequent epoxy functionalization of the terminal groups. There are several methods to achieve this epoxy functionalization, including (1) The indirect method using epichlorohydrin: Lewis acids catalyze the ring-opening reaction between the reactive end-groups of hyperbranched polymers and epichlorohydrin, yielding hyperbranched polymers with vicinal hydroxyl chlorohydrin groups [18]. Subsequent treatment with a strong base to remove HCl results in the desired product. This method is suitable for hyperbranched polymers with reactive end-groups such as hydroxyl, phenolic, or carboxyl groups that can react with epichlorohydrin. The raw materials for this method are easy to obtain, and the reaction conditions are mild and easily controllable, but some by-products can be generated during the reaction process. Zhao Ling [19] et al. employed the A_2_ + B_3_ multi-branched ring-opening polymerization method to synthesize hydroxyl-terminated hyperbranched polyesters using phthalic anhydride and epichlorohydrin. (2) The double-bond oxidation method: This method involves introducing epoxy groups by oxidizing unsaturated fatty chains with strong oxidizing agents [20]. Xia Min [21] et al. first capped the hydroxyl end-groups of hyperbranched polyesters with oleic acid to obtain hyperbranched polyesters with unsaturated fatty chain end-groups and then used the oxidizing agent meta-chloroperoxybenzoic acid to oxidize the double bonds, synthesizing hyperbranched epoxy compounds containing epoxy groups in long fatty chains. The compatibility of such hyperbranched epoxides with linear polymers can be significantly improved due to their long fat chains. (3) The multi-functional epoxy monomer proton transfer polymerization method: This method’s synthetic mechanism is similar to atom transfer radical polymerization. Specifically, multi-functional epoxy monomers undergo proton transfer polymerization catalyzed by proton transfer catalysts, yielding hyperbranched epoxy compounds in one step. T. Emrick [22] et al. systematically studied the synthesis of polyester and polyether hyperbranched polymers using proton transfer polymerization with initiators containing chlorine or bromine atoms. Compared with the aforementioned two methods, hyperbranched epoxy compounds are synthesized via a one-step polymerization reaction in the presence of a basic catalyst. However, the requirements for reaction conditions are relatively stringent.

The epoxy value is one of the important parameters affecting the physical and chemical properties of EHBPs [23]. The epoxy group is a type of medium polarity and highly reactive group. Introducing epoxy groups as end groups in the structure of HBPs endows hyperbranched polymers with unique molecular structures and multifunctionality. Based on these properties, EHBPs are extremely important modifiers in the field of composites. Jin and colleagues [24] synthesized a hyperbranched polymer with a large number of active epoxy groups at the terminals and prepared PPC/EHBP blends by melt mixing. They conducted a detailed study on the effects of the epoxy value content of EHBP on the mechanical properties, thermal properties, viscosity, and gas permeability of the PPC/EHBP blends. The results indicate that as the amount of EHBP added increases, the properties are correspondingly enhanced.

EHBPs are not only applied in polymer films and coatings, but also serve as crosslinking agents for the synthesis of other polymers. Therefore, to enhance the crosslinking degree and environmental friendliness of hyperbranched polymers, selecting a widely available and biodegradable raw material is an important research direction. Epoxidized soybean oil is a widely distributed and abundant raw material. Its chemical structure contains epoxy bonds, which enable it to undergo ring-opening reactions with alcohols, amines, and carboxylic acids to form various important compounds with different properties. In contrast, other plant oils, such as castor oil, contain C=C double bonds. These unsaturated bonds may affect the reactivity during the reaction process. Moreover, epoxidized soybean oil is distinguished by its biodegradability when compared with other raw materials used in the synthesis of epoxy-ended polymers. This study utilized biodegradable vegetable oil, specifically epoxidized soybean oil, as a raw material. It underwent ring-opening reactions with glycerol and esterification with 2,2-bis(hydroxymethyl)propionic acid to obtain epoxy soybean oil polyol (EGD) with a high hydroxyl value. Subsequently, EHBPs with varying epoxy values were synthesized by reacting EGD with different mass ratios of epichlorohydrin under strong alkaline conditions. The study investigated the effects of different epoxy values on the hydrophilicity, adhesion, rheological properties, and thermal performance of EHBPs.

## 2. Results and Discussion

### 2.1. Composition and Structure of ESO, ESOG, EGD, and EHBPs

**Figure 1:**
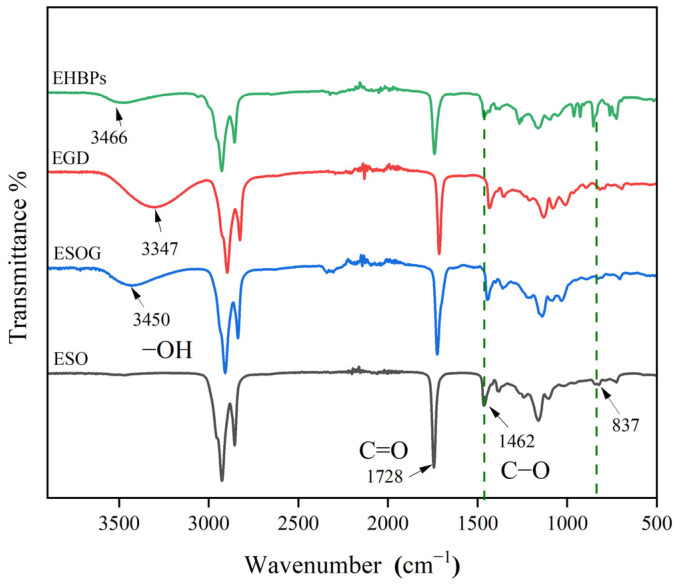
FT–IR spectra of ESO, ESOG, EGD, and EHBPs.

The FT–IR spectra of ESO, ESOG, EGD, and EHBPs are depicted in Figure 1. As shown in Figure 1, ESO exhibits a characteristic absorption peak of the epoxy group at 837 cm^−1^. In the case of ESOG, the characteristic absorption peak of the epoxy group at this location essentially vanishes, and a hydroxyl group characteristic peak emerges at 3450 cm^−1^, with no significant changes in other characteristic peaks. This indicates that ESO has successfully reacted with glycerol (GL), retaining the main structure of ESO. EHBPs exhibit a distinctive absorption peak corresponding to the epoxy group at 837 cm^−1^, and the hydroxyl group characteristic peak at 3466 cm^−1^ significantly decreases, which indicates that EGD has successfully reacted with ECH to produce epoxy soybean-oil-based epoxy-terminated hyperbranched polymers.

**Figure 2:**
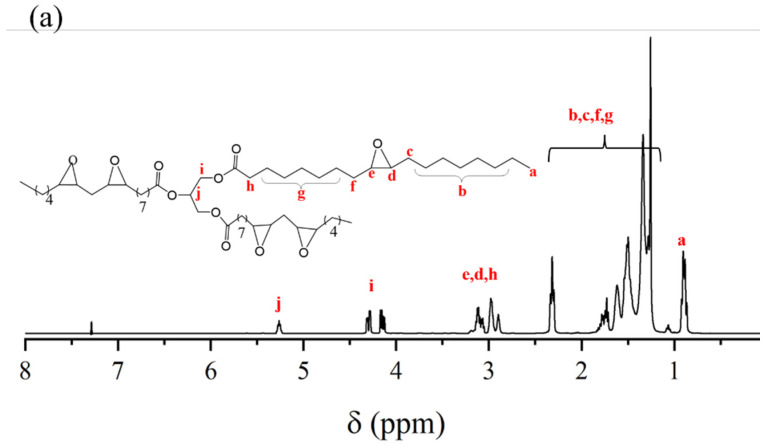

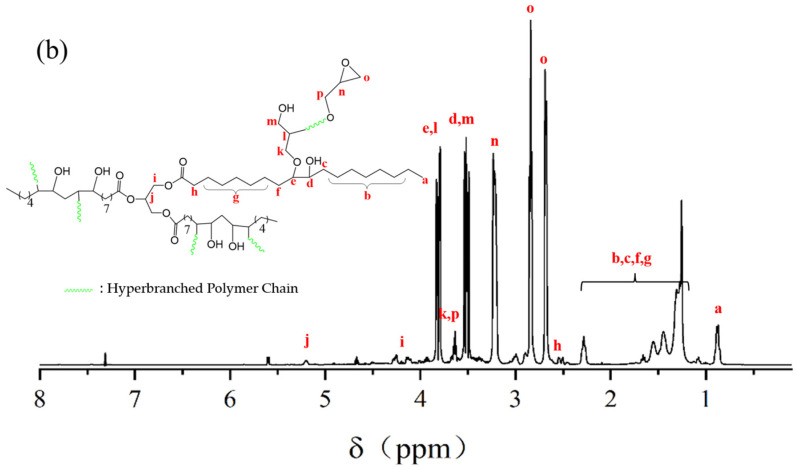
^1^H NMR of ESO (**a**) and EHBPs (**b**).

In the proton nuclear magnetic resonance (^1^H NMR) spectrum of ESO (Figure 2a), the peaks at 0.9~2.4 ppm belong to protons of −CH_2_− and −CH_3_ on the molecular chain, the chemical shift at 2.8~3.0 ppm is attributed to the hydrogen atoms attached to the epoxy group, and the chemical shift at 4.1~4.4 ppm is attributed to the hydrogen atoms on the methylene groups adjacent to the oxygen atoms in the ester groups (−OCOCH_2_ and −OCH_2_). From the ^1^H NMR spectrum of EHBPs (Figure 2b), the chemical shifts at 2.6~2.8 ppm and 3.3 ppm are attributed to the hydrogen atoms on the methylene groups adjacent to the oxygen atoms in the terminal epoxy groups (−OCH_2_ and −OCH), the signal at 3.5~3.7 ppm belongs to the hydrogen atoms on the methylene groups adjacent to the terminal hydroxyl groups, and the signal at 3.7~3.9 ppm belongs to the hydrogen atoms on the methylene groups adjacent to the oxygen atoms in the ether bonds (−OCH_2_). It is noteworthy that the signal peaks for the epoxy groups in EHBPs are much larger than those in ESO, indicating that the formed EHBPs have a large number of epoxy groups [25]. Combined with the FT–IR analysis of ESO and EHBPs, it is demonstrated that the epoxy soybean-oil-based telechelic epoxy hyperbranched polymers have been successfully synthesized.

### 2.2. Degree of Branching of EGD

**Figure 3:**
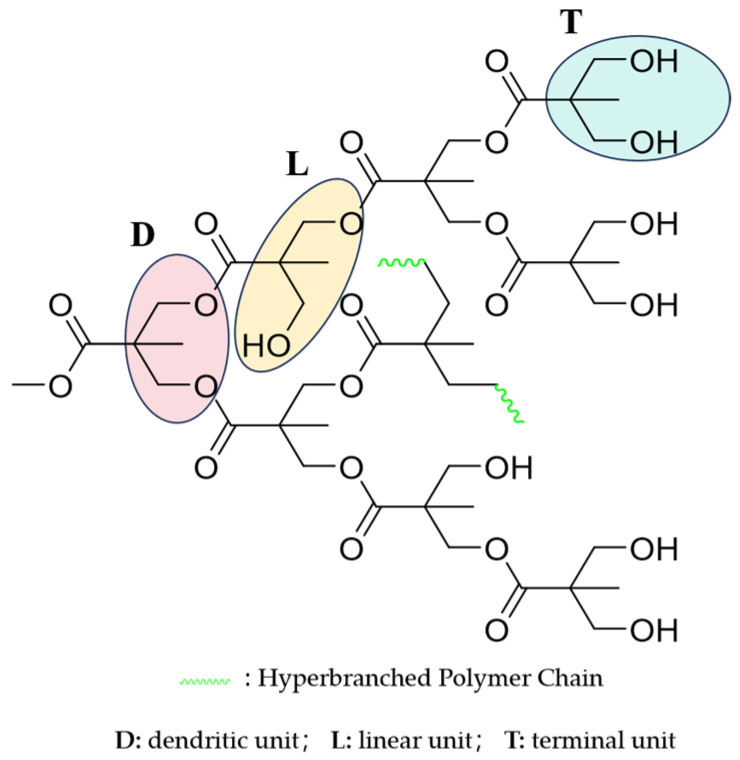
The chemical structure of EGD.

**Figure 4:**
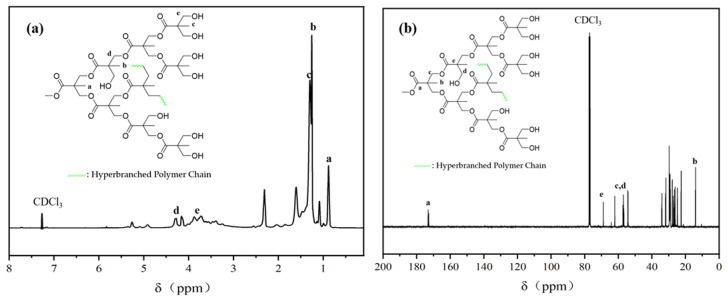
^1^H NMR (**a**) and ^13^C NMR (**b**) spectra of EGD.

Figure 3 shows the chemical structure of EGD. The branching degree of EGD can be calculated by using the relative integral area of −CH_3_ in D, L, and T, respectively. In Figure 4a, the chemical shift at 0.88 (peak a) corresponds to the proton resonance peak of D−CH_3_, the chemical shift at 1.25 (peak b) is the proton resonance peak of T−CH_3_, and the chemical shift at 1.30 (peak c) is associated with L−CH_3_. The chemical shifts between 3.60~3.99 (peak e) and 4.09~4.41 (peak d) correspond to the proton resonance peaks of −CH_2_− groups. The −CH_2_− group attached to the carbonyl group enhanced the deshielding effect due to the conjugated electron system of the carbonyl, causing the chemical shift to move toward the lower fields. Therefore, peak d is the proton resonance peak of −CH_2_− groups adjacent to the carbonyl, and peak e is the proton resonance peak of −CH_2_− groups adjacent to the hydroxyl group. In Figure 4b, the carbon resonance peak of −CH_3_ (b) appears in the high field, with chemical shifts ranging from 13.56 to 14.42; the chemical shift of the ester carbonyl carbon (a) is between 172.41 and 173.71; and the chemical shifts between 53.46 and 58.27 correspond to the carbon resonance peaks of −CH_2_− groups. Based on the above data, the relative peak areas of D−CH_3_, T−CH_3_, and L−CH_3_ in the ^1^H NMR spectrum are 1.00, 1.78, and 3.41, respectively. Substituting these values into Equation (2), EGD’s degree of branching (DB) is calculated to be 0.45, indicating that the EGD synthesized in this study is a hyperbranched polymer with high degree of branching. An increase in the branching degree can reduce the polymer’s viscosity and enhance its thermal stability. Such hyperbranched polymers with high branching can be widely applied in fields such as surface coatings and adhesives [26].

### 2.3. Molecular Weight of EHBPs

**Figure 5:**
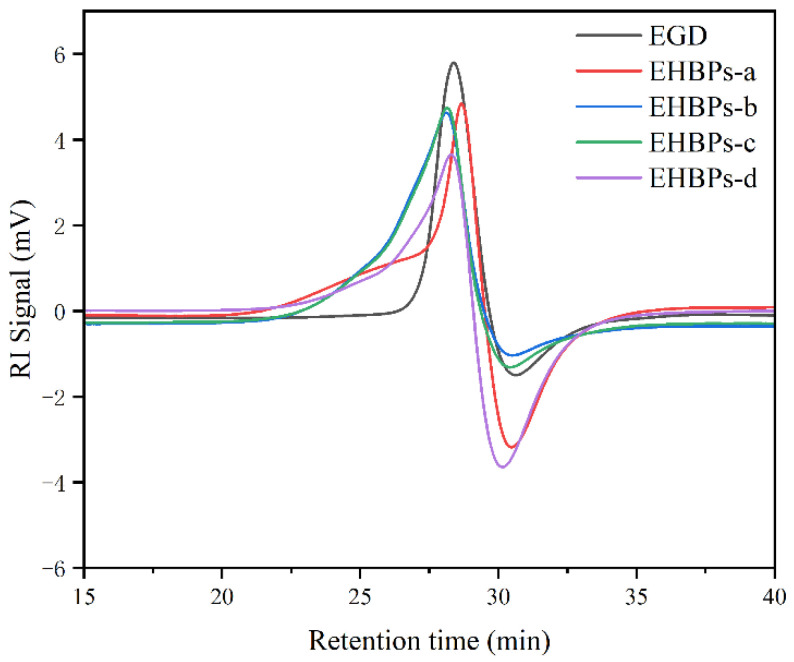
The GPC traces of EGD and EHBPs.

The epoxy value of the EHBPs was calculated using the hydrochloric acid–acetone method. As shown in Table 1, the epoxy value of the EHBPs gradually increases with the increment in ECH. Concurrently, the molecular weight of the EHBPs was determined using Gel Permeation Chromatography (GPC). Figure 5 and Table 1 reveal that the molecular weight of the EHBPs increases with the epoxy value, which is attributed to the reaction between ECH and the hydroxyl groups on the EGD molecules to form epoxy groups. An increase in the amount of ECH leads to the formation of a higher number of epoxy groups, resulting in an increase in the molecular weight of EHBPs. Additionally, it can be observed that as the epoxy value increases, the molecular weight distribution of the EHBPs becomes narrower, which may affect the performance of the polymer.

### 2.4. Rheological Properties of EHBPs

**Figure 6:**
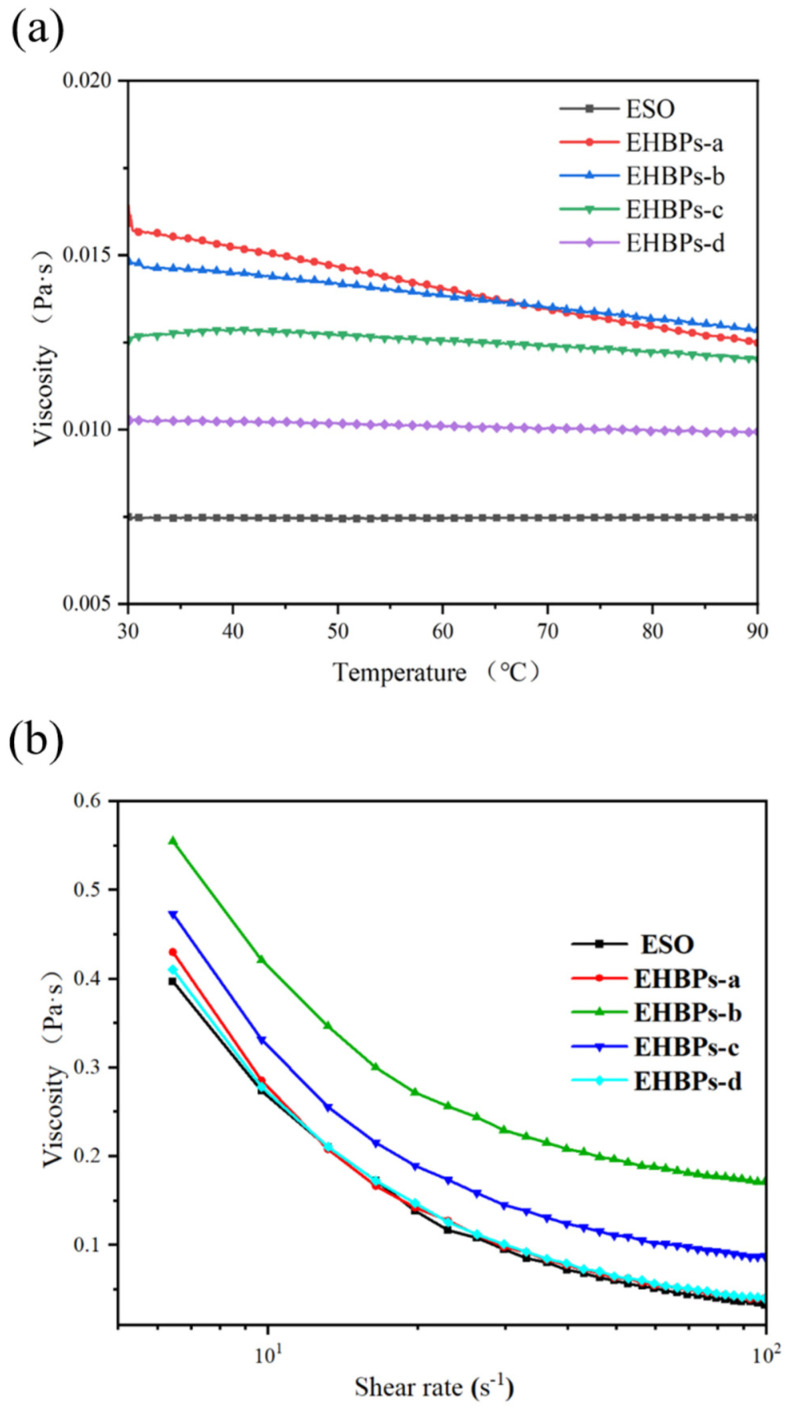
Viscosity–temperature plot of ESO and EHBPs (**a**); viscosity–shear plot (**b**).

The epoxy value of a polymer has a significant impact on its rheological properties. The figure above shows the relationship between time–viscosity and shear rate–viscosity for different EHBPs. Figure 6a shows that the viscosity of the raw material ESO is relatively low and exhibits minimal variation with increasing temperature. In contrast, the viscosity of the EHBPs decreases when the quantity of ECH incorporated increases. This is because the increase in the epoxy structure of the polymer reduces the hydrogen bonding between polymers, leading to a decrease in viscosity [27]. At a constant shear rate of 100 s^−1^, the viscosity of EHBPs-a and EHBPs-b decreases in proportion to rising temperatures. This phenomenon occurs because the thermal motion between polymer molecules becomes more intense with increasing temperature, and the chemical bonds on the molecular chains break at high temperatures, causing the viscosity of the polymer solution to decrease. From Figure 6b, it can be observed that the viscosity of both ESO and EHBPs gradually decreases with increasing shear rate, exhibiting the characteristics of pseudoplastic fluids. Due to the intermolecular entanglements and interactions within the polymer solution system, when continuously subjected to shear, the entangled structures are partially destroyed and cannot return to their original state on time. Therefore, macroscopically, this manifests as a reduction in viscosity, showing significant shear thinning behavior [28]. EHBPs-c and EHBPs-d contain a higher number of epoxy groups, which entangle with each other. This entanglement effectively decelerates the process of molecular destruction, exhibiting a slower decrease in viscosity compared to ESO. At low shear rates, the viscosity of the polymer solution decreases significantly, and as the shear rate increases, the rate at which the viscosity of the polymer solution decreases gradually diminishes. The variation in the viscosity of the EHBPs indicates that the molecular weight affects their viscosity, with higher molecular weights leading to a decrease in viscosity.

### 2.5. Thermal Performance of EHBPs

**Figure 7:**
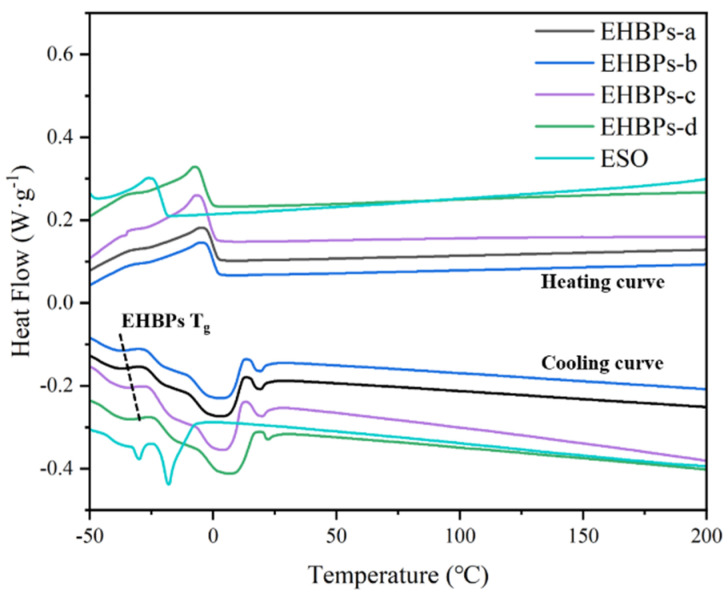
The first cooling and second heating curve graphs for ESO and EHBPs.

As depicted in Figure 7, the DSC curves of the prepared soy polyols varied markedly from each other, and the initial material, ESO, and EHBPs exhibit melting peaks around 4 °C and 20 °C. Multiple peaks for the EHBPs and ESO were ascribed to different crystalline poly-morphs that were present in the soybean oil. EHBPs have more hydroxyl groups in their chemical structure, and inter- and intramolecular hydrogen bonding causes the melting point to move to higher temperatures. As the number of epoxy groups in EHBPs rises and the hydroxyl groups fall, the melting and crystallization temperatures of these polymers tend to lower, while the melting enthalpy gradually increases. In addition, ESO and EHBPs have a distinct glass transition process, exhibiting thermodynamic characteristics similar to amorphous polymers. The glass transition temperature of EHBPs is lower than that of ESO. Table 2 presents the glass transition temperatures of EHBPs, revealing that an increase in the epoxy number correlates with a higher glass transition temperature for these polymers. The glass transition temperature of the EHBPs rises from −45 °C to −42 °C. This increase is attributed to the increased epoxy structure in EHBPs, which enhances the mobility of the polymer chains, leading to a rise in the glass transition temperature. However, the increase is insignificant, indicating that EHBPs possess stable thermal properties [29].

### 2.6. Water Contact Angle Between EGD and EHBPs

**Figure 8:**
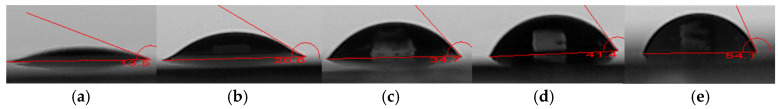
Static water contact angle diagram for EGD (**a**) and EHBPs (**b**–**e**).

The sessile drop method was employed to determine the static water contact angles of EGD and EHBPs, and the experimental results are depicted in Figure 8. The water contact angle of EGD is 14°, indicating its strong hydrophilicity, which can be attributed to the presence of a multitude of hydroxyl groups on EGD, exhibiting an extreme affinity for water. Conversely, the EHBPs exhibit a larger water contact angle, reaching up to 54°, due to the abundance of epoxy groups at their terminals, which results in weaker hydrophilicity. With the rise in epoxy value, the water contact angle of EHBPs incrementally grows, leading to a gradual decrease in their hydrophilicity.

### 2.7. Adhesion Properties of EGD and EHBPs

**Figure 9:**
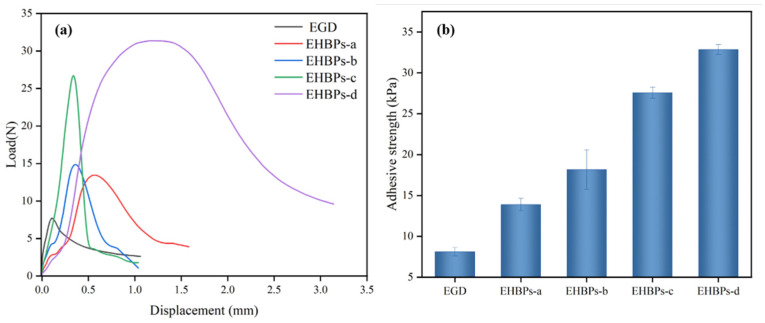
Shear curves (**a**) and adhesion strength (**b**) of EGD and EHBPs.

As shown in Figure 9a, the maximum shear forces for EGD, EHBPs-a, EHBPs-b, EHBPs-c, and EHBPs-d are 8.14, 13.47, 14.94, 26.89, and 31.42 N, respectively. Figure 9b shows the adhesion strengths to be 8.12, 13.89, 18.17, 27.57, and 32.86 kPa, respectively. It is evident that the epoxy groups can enhance the adhesion strength of the polymers, and with the increase in epoxy value, the adhesion strength of EHBPs gradually increases. This is attributed to the crosslinking reaction between EHBPs and the curing agent, forming an insoluble network structure of polymers, which enhances the cohesion and adhesion of the polymers [30]. Furthermore, as the epoxy value of EHBPs increases, the density of epoxy groups at their terminals becomes higher, which, in turn, boosts their adhesion strength. This end-epoxy hyperbranched polymer with high adhesion strength can be utilized for modifying adhesives. In addition, EHBPs with higher molecular weights and narrower molecular weight distributions exhibit greater adhesion strength, indicating that molecular weight has an impact on their adhesion properties.

## 3. Materials and Methods

### 3.1. Materials

Epoxidized soybean oil (ESO, AR) was purchased from Shandong Keyuan Pharmaceutical Co., Ltd. (Jinan, Shandong, China); sodium hydroxide (NaOH, AR) was obtained from Shanghai Aladdin Biochemical Technology Co., Ltd. (Shanghai, China); glycerol (GL, AR), tetrabutylammonium bromide (TBAB, 99%), 2,2-bis(hydroxymethyl)propionic acid (DMPA, 99%), p-toluenesulfonic acid (P-TSA, ≥97%), epichlorohydrin (99%), pyridine (99.5%), phthalic anhydride (99%), and acetone (AR) were all purchased from Shanghai Titan Technology Co, Ltd. (Shanghai, China). All materials were used as they were purchased.

### 3.2. Synthesis of End-Epoxy Hyperbranched Polymers (EHBPs)

**Figure 10:**
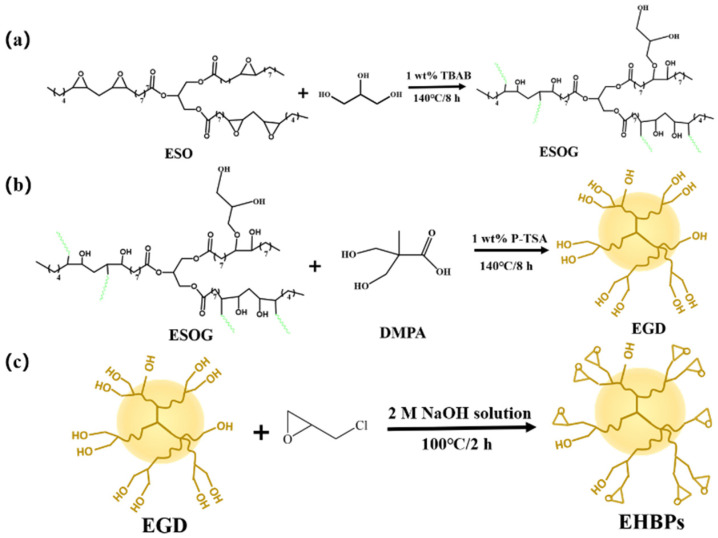
Ring-opening reaction of epoxy soybean oil (**a**); synthesis of hyperbranched polyols (**b**); synthesis of terminated epoxy hyperbranched polymers (**c**).

EHBPs were synthesized via a three-step method, as shown in Figure 10. In the initial step of the process, ESO undergoes a ring-opening reaction with GL to yield ESOR. ESOG is further esterified with DMPA in the second step to obtain the hyperbranched polyol EGD. Finally, EHBPs are synthesized through the reaction of EGD with epichlorohydrin. Specifically, ESO (20 mL), GL (20 mL), and TBAB (1 wt%) were added to a three-neck flask equipped with a reflux condenser and a stirrer. Under nitrogen protection, the mixture was heated to 140 °C and reacted until the epoxy value was less than 0.5% (Figure 10a). The hydroxyl value of the obtained ESOG was 273 ± 5 mg KOH/g. Subsequently, DMPA (10 g) and P-TSA (1 wt%) were added to the three-neck flask and reacted for 3 h (Figure 10b). The target product, EGD, was washed twice with acetone and dried in a vacuum oven at 70 °C, ensuring that the acid value of the EGD was less than 10 mg KOH/g. The hydroxyl value of the obtained EGD was 469 ± 5 mg KOH/g. Finally, EGD and epichlorohydrin in mass ratios of 1:3, 1:4, 1:5, and 1:6 were added to a three-neck flask equipped with a reflux condenser and a stirrer. Under nitrogen protection, the mixture was heated to 100 °C and reacted for 2 h, during which a 2 mol/L aqueous solution of NaOH was gradually introduced (Figure 10c). Upon completion of the reaction, the aqueous and organic layers were separated in a separatory funnel and the organic layer was washed twice with distilled water, finally being dried in a vacuum oven at 70 °C to remove excess water, resulting in a transparent pale yellow product. These synthesized EHBPs were labeled as EHBPs-a, EHBPs-b, EHBPs-c, and EHBPs-d.

### 3.3. Testing and Characterization

#### 3.3.1. Fourier Transform Infrared Spectroscopy (FT–IR)

A Thermo Fisher IS50 ATR infrared spectrometer (Waltham, MA, USA) was used to characterize ESO and EHBPs. Measurements were taken over a wavelength range of 4000 to 500 cm^−1^, with 30 scans and a resolution of 4 cm^−1^.

#### 3.3.2. ^1^H NMR Spectra

^1^H NMR spectra were tested using a Bruker Avance 400 MHz spectrometer (Ettlingen, Germany). The 5 to 15 mg samples were ultrasonically dissolved in 0.6 mL of deuterium chloroform before testing. Tetramethylsilane was used as an internal standard, and the resolution of the test frequency was set to 0.01 Hz.

#### 3.3.3. ^13^C NMR Spectra

^13^C NMR spectra were tested using a Bruker Avance 400 MHz spectrometer. The 30 mg samples were ultrasonically dissolved in 0.6 mL of deuterium chloroform before testing. Tetramethylsilane was used as an internal standard, and the resolution of the test frequency was set to 0.01 Hz.

#### 3.3.4. Gel Permeation Chromatography (GPC)

To determine the molecular weight and distribution of polymers using the Waters 1515 gel permeation chromatography instrument equipped with a refractive index (RI) detector of 2414 and a UV detector of 2489, EHBPs solutions were prepared at a concentration of 4 mg/mL, and tested at a temperature of 40 °C, with a flow rate of 1.00 mL/min, using dimethylformamide (DMF) as the solvent. The system was calibrated using narrow linear polystyrene standards with molecular weights ranging from 540 to 7.4 × 10^−5^ g/mol.

#### 3.3.5. Differential Scanning Calorimeter (DSC)

The melting and heat behavior of the ESO and EHBPs samples were recorded using a DSC25 (TA, USA) device. The procedure was as follows: Approximately 4 to 10 mg of the sample was weighed using a high-precision balance and placed into an aluminum crucible. The temperature was increased from −50 °C to 250 °C at a rate of 10 °C/min, maintained for 5 min, and then decreased back to −50 °C at a rate of 10 °C/min. The second temperature rise curve was recorded, and all experiments were conducted in an atmosphere of high-purity nitrogen gas.

#### 3.3.6. Rheological Property

Rheological behaviors were observed using the TA Instruments HR10 rotational rheometer. A 2000 mg/L solution of EHBPs was prepared and placed between plates with a diameter of 60 mm. The relationship between viscosity and temperature was measured at a frequency of 100 s^−1^ within the range of 30 °C to 90 °C. Similarly, the relationship between viscosity and shear rate was measured at a temperature of 60 °C over a range of 0 to 100 s^−1^ (γ = 0.5%).

#### 3.3.7. Epoxy Value Measurement

According to the Chinese National Standard GB/T 1677-2023 [31], the epoxy value of the reaction product was determined using the hydrochloric acid–acetone method. The epoxy value was calculated as shown in Equation (1):

(1) $$E=\frac{\left(v_{0}-v_{1}\right)*c}{10m}$$

where *v*_0_ is the volume of NaOH/ethanol solution consumed in the blank control group (mL); *v*_1_ is the volume of NaOH/ethanol solution consumed when determining the epoxy value of the sample (mL); *m* is the mass of the sample (g); and *c* is the concentration of the NaOH/ethanol solution (mol/L).

#### 3.3.8. Degree of Branching

The degree of branching (DB) of the EHBPs was calculated using their ^1^H NMR spectrum, as shown in Equation (2):

(2) $$DB=\frac{I_{D}+I_{T}}{I_{D}+I_{T}+I_{L}}\times 100$$

where *I_D_* is the sum of the integrated intensities of the absorption peaks for the branching units, *I_T_* is the sum of the integrated intensities of the absorption peaks for the terminal units, and *I_L_* is the sum of the integrated intensities of the absorption peaks for the linear units.

#### 3.3.9. Water Contact Angle Measurement

Samples of EGD and EHBPs were evenly coated onto microscope slides and allowed to remain at room temperature for 24 h to form a film. The static contact angles of the samples were determined with a contact angle meter model JH-901A (Jinhua Yi (Beijing) Technology Co., Ltd., Beijing, China), employing deionized water as the testing liquid, with measurements taken at three arbitrary locations on each sample. The mean value of the measurements was designated as the static contact angle of the sample.

#### 3.3.10. Adhesion Measurement

The lap shear adhesion tests of EGD and EHBPs were conducted using a universal testing machine, model LD24.104 (Lishi (Shanghai) Instruments Co., Ltd., Shanghai, China). The sample was uniformly applied onto the upper surface of a glass substrate measuring approximately 24 mm × 70 mm, and then another glass surface was placed on top. A 1 kg weight was applied for 1 min to ensure proper adhesion. The test specimen was fixed in a tensile clamp, and the test was conducted at a stretching rate of 20 mm/min. The maximum load was recorded, and the adhesion strength was calculated using the formula F/S, where F is the maximum load and S is the bonding area.

## 4. Conclusions

In this study, epoxy-terminated hyperbranched polymers (EHBPs) were synthesized from epoxidized soybean oil. Meanwhile, the influences of the epoxy value of these polymers on their performance characteristics were investigated. The degree of branching of EGD was calculated to be 0.45 using NMR spectroscopy. The rheological properties, thermal properties, water contact angle, and adhesion strength of four types of EHBPs were examined. The results indicate that EHBPs with higher epoxy values and molecular weights exhibit reduced hydrophilicity and greater adhesion strength, and their viscosity decreases more slowly with increases in temperature and shear rate. The epoxy value has a minimal impact on the thermal properties of EHBPs, which maintain stable thermal performance. These hyperbranched polymers with a high degree of branching, significant adhesion strength, and excellent thermal stability offer new perspectives for the development of surface coatings and adhesives.

## Figures and Tables

**Table 1:** Epoxy value, M_n_, and PDI of ESO, EGD, and EHBPs.

Samples	n (EGD): n (ECH)	Epoxy Value (mol/100g)	M_n_ (Da)	PDI
ESO	-	0.375	1050	1.3
EGD	-	-	2100	1.04
EHBPs-a	1:3	0.73	2650	1.37
EHBPs-b	1:4	0.79	2900	1.22
EHBPs-c	1:5	0.82	2950	1.25
EHBPs-d	1:6	0.89	3300	1.20

**Table 2:** Thermal properties of EHBPs.

Samples	T_m_/°C		ΔH_m_/(J·g^−1^)	T_c_/°C	T_g_/°C
	1	2			
EHBPs-a	3.28	18.65	6.68	−4.29	−45.70
EHBPs-b	3.46	19.19	6.96	−4.21	−44.97
EHBPs-c	3.54	19.48	7.82	−5.97	−43.56
EHBPs-d	3.76	20.33	8.07	−6.31	−42.14

## Data Availability

The original contributions presented in this study are included in the article. Further inquiries can be directed to the corresponding authors.
